# Methods to analyze SNARE-dependent vesicular fusion events that regulate autophagosome biogenesis

**DOI:** 10.1016/j.ymeth.2014.11.005

**Published:** 2015-03-15

**Authors:** Kevin Moreau, Claudia Puri, David C Rubinsztein

**Affiliations:** Department of Medical Genetics, Cambridge Institute for Medical Research, University of Cambridge, Cambridge CB2 0XY, UK

**Keywords:** Autophagy, ATG16L1, mATG9, Homotypic/heterotypic fusion

## Abstract

Autophagy is an important catabolic pathway that preserves cellular homeostasis. The formation of autophagosomes is a complex process requiring the reorganization of membranes from different compartments. Here we describe methods to analyze SNARE-dependent vesicular fusion events involving the homotypic and heterotypic fusion of autophagosome precursor structures. These two steps are essential for the maturation of small single-membrane autophagic precursors containing ATG16L1 and mATG9 proteins into double-membrane autophagosomes. The techniques described in this review are mostly based on live cell imaging, microscopy, and biochemistry using an in vitro fusion assay, and should help researchers to study autophagosome biogenesis.

## 1 Introduction

(Macro) autophagy is a critical clearance pathway for organelles and long-lived proteins, including intracytoplasmic aggregate-prone proteins that cause many neurodegenerative diseases, such as huntingtin in Huntington’s disease, and tau in Alzheimer’s disease [1]. Autophagosomes are double-membraned structures that engulf portions of cytoplasm and ultimately fuse with lysosomes, where their contents are degraded. The first recognizable structures in the pathway are cup-shaped phagophores, whose edges extend and fuse to form autophagosomes [2,3]. The ATG5–ATG12/ATG16L1 complex regulates the initiation of phagophore formation, while phosphatidylethanolamine-conjugated ATG8/LC3 (LC3-II) mediates the elongation and fusion of the phagophore edges to form autophagosomes [2,3]. The ATG5–ATG12/ATG16L1 complex decorates the phagophore and dissociates after completion of autophagosome formation, while LC3-II is localized both to the phagophore and fully formed autophagosomes.

Clathrin-mediated endocytosis regulates autophagy by enabling membrane delivery to ATG5/ATG12/ATG16L1-positive phagophore precursor vesicles (LC3-negative), which mature to form phagophores (ATG16L1-positive and LC3-positive), and subsequently into autophagosomes (ATG16L1-negative and LC3-positive) [4]. The ATG16L1-positive/LC3-negative phagophore precursors undergo homotypic fusion events that increase their size and enhance their ability to acquire LC3-II [5]. These fusion events are mediated by SNAREs (an acronym derived from “SNAP (Soluble NSF Attachment Protein) REceptors”), including VAMP7, Syntaxin 7, Syntaxin 8 and Vti1B [5]. The maturation of the ATG16L1-positive precursors also requires VAMP3-mediated fusion with mATG9-positive vesicles [6]. Interestingly, VAMP3 depletion does not affect ATG16L1 homotypic fusion [6]. Currently, it is not clear if these homotypic and heterotypic fusion events are sequential or parallel. However, these data reveal that the SNARE-dependent fusion of distinct vesicles containing different autophagy proteins is required for optimal autophagosome biogenesis. Interestingly, these fusions occur prior to the phagophore stage.

**Fig. 1:**
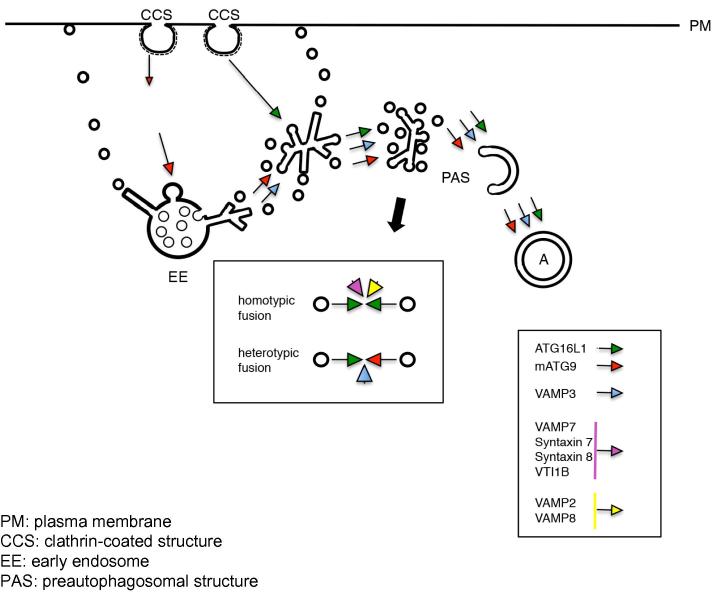
Schematic diagram of mATG9 and ATG16L1 itineraries.

We observed that ATG16L1 and mATG9 both traffic via the plasma membrane. However, they are located in distinct clathrin-coated pits and are internalized and trafficked through largely different routes [6]. mATG9 follows the transferrin receptor pathway through early endosomes and recycling endosomes, whereas ATG16L1 reaches the recycling endosomes but has negligible association with early endosomes. The two different pools of vesicles carrying mATG9 and ATG16L1 coalesce in the recycling endosomes at a stage prior to phagophore formation [6]. If one inhibits this process by knocking down VAMP3, then autophagosome formation is impaired (Fig. 1).

We recently identified PICALM (CALM; phosphatidylinositol binding clathrin assembly protein), recently associated with Alzheimer’s disease, as an important regulator of both the homotypic fusion and the heterotypic fusion of autophagic precursors [7]. This can be attributed to its role as a clathrin adaptor which mediates the endocytosis of various SNAREs, including VAMP2, VAMP3 and VAMP8 [7]. In CALM knockdown cells, VAMP2 (a newly identified SNARE involved in autophagosome formation) is no longer present on ATG16L1 vesicles resulting in impaired homotypic fusion of ATG16L1 vesicles; and VAMP3 is no longer associated with mATG9, which impairs the heterotypic fusion of ATG16L1 and mATG9 vesicles (Fig. 1 – in the box) [7]. The downregulation of autophagy when CALM expression was modified was also associated with a decrease in the clearance of tau, a autophagy substrate which is a key hallmark of Alzheimer’s disease [7].

In this review, we will describe methods to analyze the homotypic fusion of ATG16L1 vesicles and the heterotypic fusion of ATG16L1 and mATG9 vesicles. This is mostly based on live cell imaging and an in vitro fusion assay, which will be the methods presented below.

## 2 Protocols

### 2.1 Homotypic fusion of ATG16L1 vesicles

#### 2.1.1 Live cells imaging

Seed cells (HeLa) into a 35 mm MatTek dish (MatTek Corporation) at 1 × 10^5^ cells per dish. Transfect cells with GFP–ATG16L1 plasmid 24 h before imaging using TransIT2020 transfection reagent (Mirus), according to the manufacturer’s protocol. We use 300 ng per 6 well dish to minimize overexpression artifacts. The day after transfection, autophagy can be stimulated by amino acid- and serum-starvation in Hanks balanced salt solution (HBSS; Sigma) for 1–4 h. This greatly facilitates the analysis, since it induces the production of autophagic precursors, facilitating their detection by microscopy. The dish is then mounted on a live cell imaging system (Zeiss LSM710) with a CO_2_ incubation chamber. Acquisition is done at maximal camera speed for 5–10 min with minimal exposure in order to avoid photobleaching. A movie is created and analyzed manually to score homotypic fusion between ATG16L1 vesicles. Fused vesicles are scored when two vesicles are in contact for more than 10 frames. Note that one should exclude any cells with obviously clumped ATG16L1 structures, which are likely artifacts.

#### 2.1.2 Fixed cell imaging

Homotypic fusion between ATG16L1 vesicles increases their size that can be measured by microscopy. Place 13 × 13 mm square coverslips into the bottom of each well of a 6 well plate and seed cells into these plates at 1 × 10^5^ cells per well. The confluency of the cells at this stage is critical, as too high a density will preclude automatic counting of vesicles, since the microscope will be not able to focus properly on the cells. As for live cells imaging, the day after seeding, autophagy can be stimulated by treating the cells with HBSS for 1–4 h to induce the production of autophagic precursors, facilitating their detection by microscopy [4]. Fix the cells with 4% paraformaldehyde in PBS for 5 min. Wash the cells three times with PBS. Mount the cells on slides using antifade reagents (ProLong, Invitrogen). If you have access to the Cellomics ArrayScan VTI HCS Reader, use the Spot Detector Bioapplication protocol version 3 to count the number of vesicles per cell and to measure the size of the vesicles. If you do not have an automatic microscope, ImageJ is an alternative. To measure the size of vesicles using ImageJ, take high-resolution pictures of your cells using a confocal microscope. Open your pictures in ImageJ, split the channels, invert the picture to have white background, adjust the threshold to take off the background, apply the threshold, open analyze particles in analyze option, apply and you have a table with the number of vesicle and their size. We used both methods to assess ATG16L1 or autophagic vesicle sizes. The Thermo Scientific Cellomics ArrayScan VTI HCS has a resolution of around 400 nm whereas quantification using ImageJ from confocal pictures has a resolution of ∼150 nm. For these experiments, one should inspect cells prior to automated imaging to ensure that any clumped ATG16L1 is minimal, as should be the case if cells are transfected with low amounts of the plasmid, as described above.

#### 2.1.3 In vitro fusion assay

This assay was modified from the method described by Barysch et al. [8] for analyzing endosome docking/fusion and sorting/budding, based on labeling of endosomes by endocytotic uptake with fluorescent cargoes. We adapted this protocol to describe and measure homotypic fusion of autophagic precursors. Two different pools of HeLa cells (1 × 10^6^) were transfected for 20 h with either mStrawberry–ATG16L1 or GFP–ATG16L1. Two postnuclear supernatants (PNS) were obtained by mechanical disruption. Transfected HeLa cells were scraped in 500 μl HB buffer (Homogenization Buffer: 250 mM sucrose and 3 mM imidazole, pH 7.4 (with HCl) and proteases inhibitors) and disrupted by vortexing for 1 min in the presence of glass beads (1:1) (acid-washed 425–600 μm). The mechanically disrupted cells were centrifuged for 15 min at 1200 rcf at 4 °C. The supernatant was collected and 20 μl were mixed for 60 min in the presence of an ATP regenerative system (10×: DHM buffer: 625 mM HEPES, 75 mM magnesium acetate and 10 mM DTT (pH 7.4, with KOH), 1 M potassium acetate, 100 mM ATP, 800 mM creatine phosphate, 4 mg ml^−^^1^ creatine kinase (=3200 U ml^−^^1^), 250 mM glucose and 100 mM ATP) at 37 °C (a control sample was left on ice) in low adhesive Eppendorf tubes in a total volume of 30 μl. After the reaction, the samples were fixed with 2% paraformaldehyde in PBS for 15 min (1:1 with 4% PFA), centrifuged to remove the fixative (13,000 rpm 5 min), re-suspended in distilled water and mounted with Mowiol® 4-88 with DAPI on glass coverslips for confocal observation. DAPI staining was used assist focusing on the coverslip.

### 2.2 Heterotypic fusion of ATG16L1 and ATG9A vesicles

#### 2.2.1 In vitro fusion assay of ATG16L1 and mATG9 vesicles

As described above, the quantification of homotypic fusion between ATG16L1 and ATG16L1 vesicles is an important parameter to enable characterization of this early event of autophagosome biogenesis. The approach described above can be easily applied to measure heterotypic fusion of ATG16L1/mATG9 carrying vesicles. HeLa cells are transfected for 20 h with mStrawberry–ATG16L1 or mATG9–GFP and we followed the same protocol described above for homotypic fusion in Section 2.1.3.

#### 2.2.2 Colocalisation of ATG16L1 and mATG9 vesicles

HeLa cells silenced or not for VAMP3 for 72 h using siRNAs (100 nmol of SMARTpool_L-011934-00 from Dharmacon; using Lipofectamine2000 reagent from Invitrogen) were transfected with mStrawberry–ATG16L1 for 20 h (using TransIT2020 reagent from Mirus) and grown on coverslips at confluencies of 25%. The cells were then fixed in 4% paraformaldehyde for 5 min and then permeabilised with 0.1% Triton. Fixed cells were incubated with anti-mATG9 antibody (ABCAM; Rabbit monoclonal [EPR2450(2)]), 1% BSA in PBS, followed by secondary antibody in the same buffer. Alternatively, HeLa cells transfected with mStrawberry–ATG16L1 for 20 h were left at 37 °C or placed at 18 °C for 4 h. Fixed cells were incubated with anti-mATG9 antibody (ABCAM), 1% BSA in PBS, followed by secondary antibody in the same buffer. A Zeiss LSM710 confocal microscope was used for fluorescent confocal analysis. All confocal images were taken with a 63× oil-immersion lens. Volocity software (PerkinElmer) was used for colocalisation analysis using Pearson’s Coefficient (or Manders’ Coefficient) and processing of confocal images. The Pearson’s Coefficient was used to measure the correlation between the signals from two different markers/proteins. The Manders’ Coefficient was used to quantify the localisation of protein A in compartment B under different conditions. A minimum of 20 cells were examined each condition. All experiments are repeated at least three times. The background was fixed for all within-experiment analyzes (for Mander’s).

#### 2.2.3 Live imaging of ATG16L1 and mATG9 fusion

HeLa cells were seeded on MatTek Petri dish (MatTek, Ashland, MA, USA) at a density of approximately 1.5 × 10^5^ cells per dish and transfected with mATG9–GFP and mStrawberry–ATG16L1. After 20 h, the cells were placed in HBSS (Ca+Mg^+^) with HEPES, after which they were imaged immediately at 37 °C. Five movies of 5 min were recorded for each experiment. Imaging was performed on a Zeiss Axiovert 200 M microscope with a LSM 710 confocal attachment using a 63× 1.4 NA Plan Apochromat oil-immersion lens. The fusion events between ATG16L1 and mATG9 vesicles were observed in the different movies.

## 3 Results and discussion

### 3.1 Homotypic fusion

#### 3.1.1 Live cell imaging

**Fig. 2:**
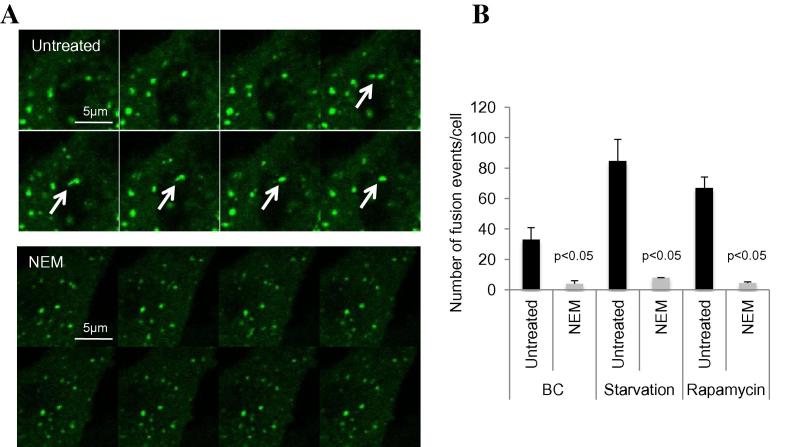
Homotypic fusion between ATG16L1 vesicles *in vivo*.

Live cell imaging is probably the best method to observe a defect in the homotypic fusion of ATG16L1 vesicles and it avoids potential artifacts due to cell preparation such as fixation or lysis. We identified SNAREs, which primarily mediate vesicle fusion, as important determinants of this process (VAMP7, Syntaxin7, Syntaxin8 and Vti1B). Using a SNARE inhibitor called NEM (N-Ethyl maleimide), that inhibits an ATPase involved in SNAREs-dependent fusion, we observed a clear defect in the homotypic fusion of ATG16L1 vesicles (Fig. 2). Moreover, when we stimulated autophagy by rapamycin treatment or by amino acid and serum starvation, we observed an increase in the rate of homotypic fusion (Fig. 2).

#### 3.1.2 Fixed cells imaging

**Fig. 3:**
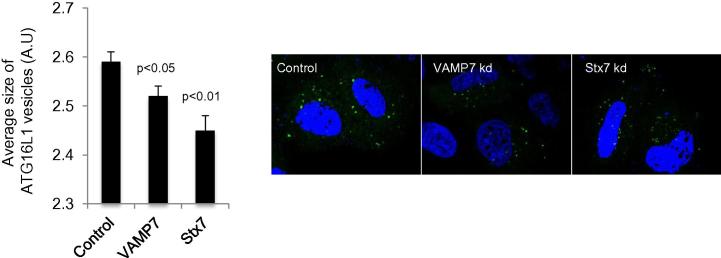
Size of ATG16L1 vesicles in VAMP7 and Syntaxin7 knockdown cells.

To complement the live cell imaging strategy, we measured the sizes of ATG16L1 vesicles by microscopy on fixed samples to provide additional information regarding the maturation of ATG16L1 vesicles. Indeed, the homotypic fusion of ATG16L1 vesicles increases their size, which can be easily measured using post-acquisition software, such as the one included in the Cellomics ArrayScan VTI HCS Reader: Spot Detector Bioapplication protocol version 3. In the example below, the size of ATG16L1 vesicles decrease in VAMP7 or Syntaxin 7 (Stx7) knockdown cells (Fig. 3), where fusion is abrogated.

#### 3.1.3 In vitro fusion assay

**Fig. 4:**
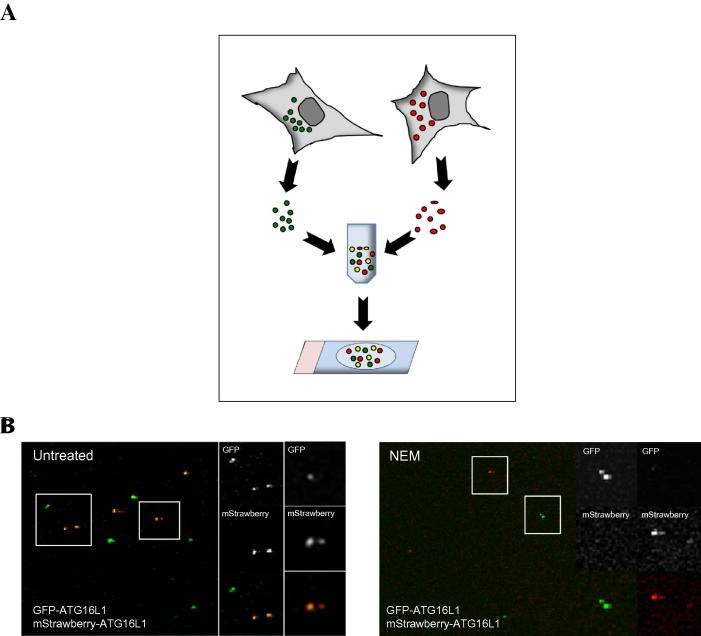
Homotypic fusion between ATG16L1 vesicles *in vitro*.

The last method used to study the homotypic fusion of ATG16L1 vesicles is an in vitro fusion assay between different fluorescent pools of ATG16L1 vesicles. In this assay, the fused vesicles appear yellow (as the consequence of green and red colocalization) that can be easily quantified (Fig. 4). In the example below, we used the SNARE inhibitor NEM to show a decrease in yellow vesicles (fused vesicles), indicating a defect in the homotypic fusion of ATG16L1 vesicles (Fig. 4).

### 3.2 Heterotypic fusion

#### 3.2.1 In vitro fusion assay of ATG16L1 and mATG9 vesicles

**Fig. 5:**
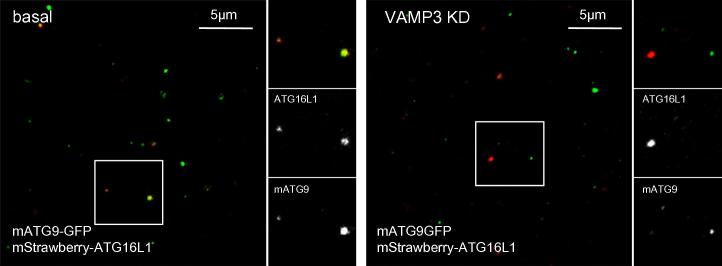
Heterotypic fusion between ATG16L1 and mATG9 vesicles *in vitro*.

The method described above can be also be used to analyze the heterotypic fusion between ATG16L1 and mATG9 carrying vesicles. Here we show how this method can be used to analyze the heterotypic fusions between ATG16L1 and mATG9 vesicles and provide a control where the critical SNARE, VAMP3, was depleted. Silencing of VAMP3 led to a strong decrease of the colocalisation between the green and red vesicles (Fig. 5).

#### 3.2.2 Colocalisation by immunofluorescence of ATG16L1 and mATG9 vesicles

**Fig. 6:**
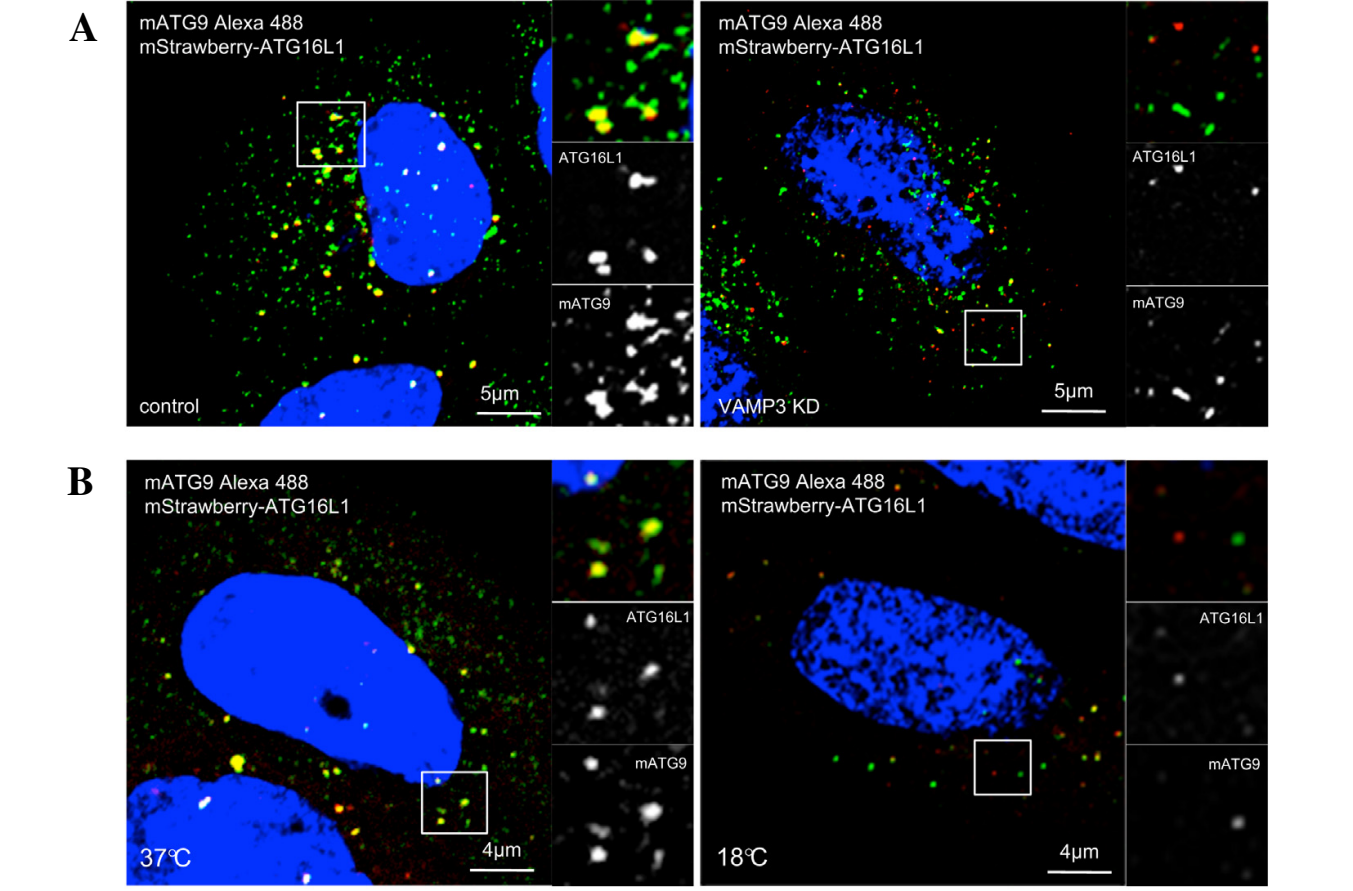
Colocalisation by immunofluorescence of ATG16 and ATG9 vesicles.

The colocalisation between ATG16L1 and mATG9 can also be measured in whole cells by classical immunofluorescence and confocal analysis. Loss of interaction between the ATG16L1 and mATG9 compartments was observed again in cells where VAMP3 was silenced, as well as in cells placed for 4 h at 18 °C, where the membrane traffic between the early endosomes and recycling endosomes was inhibited (Fig. 6).

#### 3.2.3 Live imaging of ATG16L1 and mATG9 fusion

Once we identified VAMP3 as a key element regulating the heterotypic fusion between ATG16L1 and mATG9 structures, we studied cells by live imaging. In basal conditions, one can observe vesicles carrying ATG16L1 fusing with those with mATG9. In the absence of VAMP3, the two pools of vesicles approach each other but never fuse. These experiments explain the decreased colocalisation between ATG16L1 and mATG9 that was observed using the methods described above.

## 4 Conclusion

In this review, we describe methods to study early steps that are important for autophagosome formation – the homotypic fusion of ATG16L1 vesicles and the heterotypic fusion between ATG16L1 and mATG9 vesicles. These two steps are crucial for proper maturation of autophagic precursors into phagophores [5,6] and involve SNAREs. Interestingly, different SNAREs regulate the homotypic and the heterotypic fusion. VAMP7 and VAMP2 are involved in the homotypic fusion of ATG16L1 vesicles but not in the heterotypic fusion between ATG16L1 and mATG9 vesicles, whereas VAMP3 is involved only in the heterotypic fusion [5,6]. Even if we do not know the sequential relationships of the homotypic versus the heterotypic fusions (it could happen simultaneously), they represent important events. The fact that different SNAREs are involved suggest that different compartments participate and coalesce during autophagosome biogenesis. This concept is consistent with the publications on autophagosome formation involving different intracellular compartments such as endosomes, recycling endosomes, Golgi, exocyst, endoplasmic reticulum and mitochondria [4,6,9–12]. Obviously, these methods have to be used in combinations with other techniques in order to get a better insight about the role of a protein in autophagosome formation. These methods include the measurement of LC3-II by western blot, electron microscopy, autophagy substrate clearance assays, well described in many publications [13–15].
